# Health Insurance Notes

**Published:** 1923-05

**Authors:** 


### NATIONAL HEALTH INSURANCE NOTES.

### THE DEPOSIT CONTRIBUTOR MUST GO.

INTEREST in the case of the Deposit Contributor appears never to be sustained for a sufficient length of time for anything to get done. We do not nowadays hear so much of his "unfortunate lot," and probably with good reason. It is very doubtful if the Deposit Contributor is a bad life in general; it is more probable that he or she is an insured person too lazy or indifferent, or even contemptuous, to join an Approved Society. It is more than probable that a few of the larger societies would take over the whole of them, in proportion to their membership; and all those willing to be so absorbed would be at once provided for. As to the remainder, and those new entrants from time to time who would refuse to apply for membership in, or accept any assignment to, an approved society, it is certainly not worth while providing expensive administrative machinery for dealing with their current accounts. Their contributions and those of their employers could well go into a common pool, out of which suitable reserves might be provided, in whole or in part, in the event of a belated acceptance of the benefits of insurance.

### WHY GIVE AN EXEMPT PERSON BENEFIT?

There is really no good case for providing the exempt person with medical benefit. The provision involves expense and complication of the administrative machine, and the case for it rests probably on no better theory than that the employer is entitled to expect that his contributions go to the benefit of his employé and not into a common pool. The fact is that employers in general may grunt about having to pay contributions for exempt persons, but are entirely unconcerned as to what is done with the money when it finds its way into the National Health Insurance Fund. When an employed person has, of his own volition, obtained exemption from the payment of contributions on the ground that he is otherwise adequately provided for, it seems a little absurd to endeavour to thrust some form of benefit on him. The medical benefit for exempt persons might well be wiped out, and a great many of these persons would be very glad not to be bothered about a benefit which they do not want, and of which they will not take advantage.

### VACCINATION.

The question of the panel doctor's liability in regard to the vaccination of his insured patients is one which ought to be put on a more satisfactory footing before the next settlement with the panel doctors is made. It is clear that he is at present under an obligation to vaccinate any insured person on his list who applies to him, unless he is prepared to take the responsibility of advising that vaccination is unnecessary. And yet the insured person may equally apply to the public vaccinator, and if the panel doctor happens to be the public vaccinator he is not debarred from receiving payment from public funds in that capacity. Admittedly the problem is difficult, and it may be that the only satisfactory solution is to exclude the service from the panel doctor's obligation.

### THE PRIVATE DOCTOR'S PECCADILLOES.

A particularly well-informed correspondent invites our attention to the fact that complaints by patients against doctors attending them in their private capacity are all too common, and he asks what is to be done when the charge is not sufficiently serious to bring to the notice of the General Medical Council. It is an interesting reminder of the fact that inefficient doctors have not been created by the Insurance Medical Service, but that the machinery of that service has merely served to bring to light their misdeeds. Frankly, we do not know what is to be done in the case of the private doctor beyond giving him up if another doctor is available.

### PROPER DRESSINGS.

It is just by such simple everyday things as bandages and dressings that the panel system is very largely judged. The introduction into the British Pharmaceutical Codex, at the instance of the Ministry of Health, of standards for surgical dressings, and the embodiment of these in the Insurance Tariff, are matters on which the Pharmaceutical Society and the Ministry are to be congratulated. The requirement that all cotton wools, gauzes, lints and standard dressings are to be supplied in original sealed packets is particularly valuable.

### THE RANGE OF MEDICAL SERVICE.

An important question has just been decided by a Court of Referees who held an inquiry under Article 38 of the Medical Benefit Regulations. The inquiry was held in relation to the question whether the taking of blood (venous) as a preliminary to the carrying out of the Wassermann Test for Syphilis was within the range of medical service for the insured—in other words, whether the service was one which, consistently with the best interests of the patient, could be properly undertaken by a general practitioner of ordinary competence and skill. The service was in fact performed by an insurance doctor whose competence was in no way in question. But the Local Medical Committee and the Insurance Committee contended that, although the operation was not difficult or dangerous, it was contrary to the best interests of the patient that a general practitioner should perform it where there existed facilities for treatment in the shape of efficient clinics or other institutions, equally convenient for the insured person.

### THE REFEREES' DECISION.

The Referees expressed the view that such a contention would strike at the whole basis of medical practice under the Insurance Acts, and would whittle away the duties of a general practitioner in a way inimical not only to the policy of the Insurance Acts, but also to the best interests of the profession. "' We should deprecate," they say, " any narrowing of the range of medical service by setting up a standard in which the risk of failure in an operation not difficult or dangerous was an element." They decided that the operation was within the range of service for the insured, and we are satisfied that the reasons governing the decision are eminently sound.

